# Expression of concern: MicroRNA-379 inhibits the proliferation, migration and invasion of human osteosarcoma cells by targeting EIF4G2

**DOI:** 10.1042/BSR-20160542_EOC

**Published:** 2020-08-21

**Keywords:** EIF4G2, Invasion, MicroRNA-379, Migration, Osteosarcoma, Proliferation

The Editorial Office has been made aware of potential issues surrounding the scientific validity of this paper, hence has issued an expression of concern to notify readers whilst the Editorial Office investigates.

